# Supplementary material for: Interdependent IL-7 and IFN-γ signalling in T-cell controls tumour eradication by combined α-CTLA-4+α-PD-1 therapy
Source: Nat Commun. 2016 Aug 8;7:12335. doi: 10.1038/ncomms12335 (PMC4979067; doi:10.1038/ncomms12335)
Supplement: Supplementary Information — Supplementary Figures 1-7 [file ncomms12335-s1.pdf]

Supplementary Fig. 1

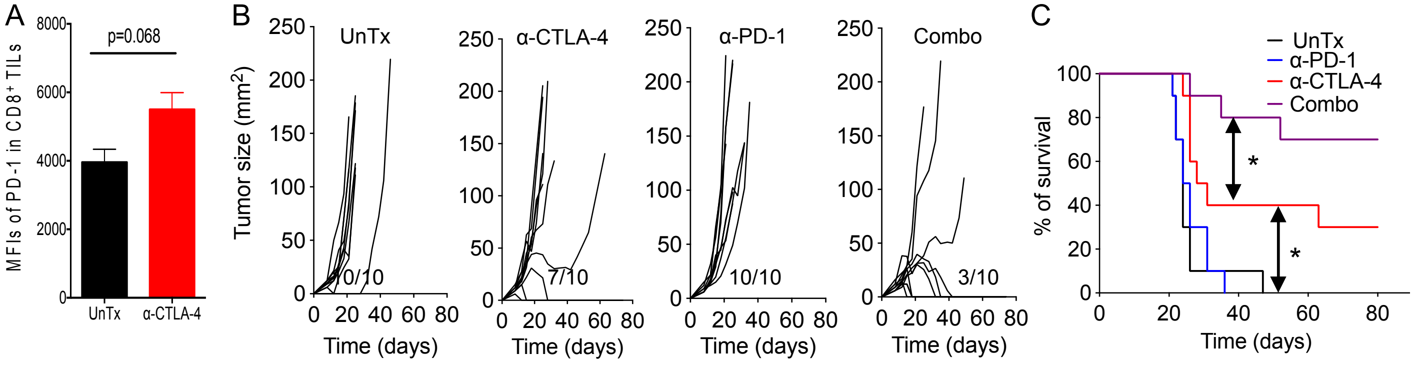

Supplementary Figure 1. PD-1 expression on CD8+ TILs and synergistic effects of combination therapy of α-CTLA-4 and α-PD-1 in RENCA tumor rejection. (A) PD-1 expression on CD8+ TILs isolated from MB49 tumor-bearing mice untreated (UnTx) or treated with α-CTLA-4. (B-C) Mice bearing 8 days palpable RENCA tumors were left untreated (UnTx) or treated with α-CTLA-4, α-PD-1, or combination of both (Combo). (B) Individual tumor growth. Tumor size is presented as length x width (mm^2) and ratios of tumor-growing mice in each group are shown in the insets on each panel. (C) Survival curves are shown from one representative experiment. Figures represent 1 of 3 independent experiments. *, $p<0.05$ by Log-rank (Mantel-Cox) test.

Supplementary Fig. 2

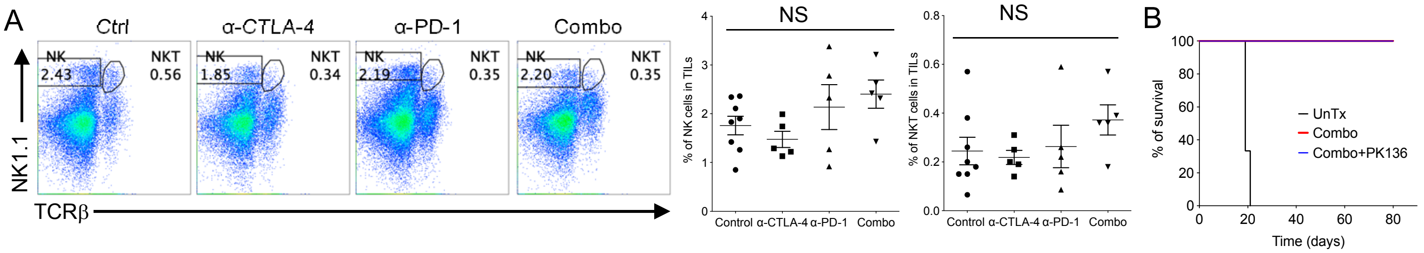

Supplementary Figure 2. NK and NKT cells are not required for rejection of MB49 tumor by combination therapy. (A) Proportions of NK and NKT cells in isolated TILs as described in **Figure 3**. Scatter plots indicate pooled results from multiple mice. (B) Control mice or mice depleted of $NK1.1^{+}$ cells (PK136) were treated with combo and mouse survivals are presented. Data are means ± SEM of 5 mice in each group. NS, no statistical significance by one-way ANOVA with Bonferroni's *post hoc* test. Data are representative of two independent experiments.

Supplementary Fig. 3

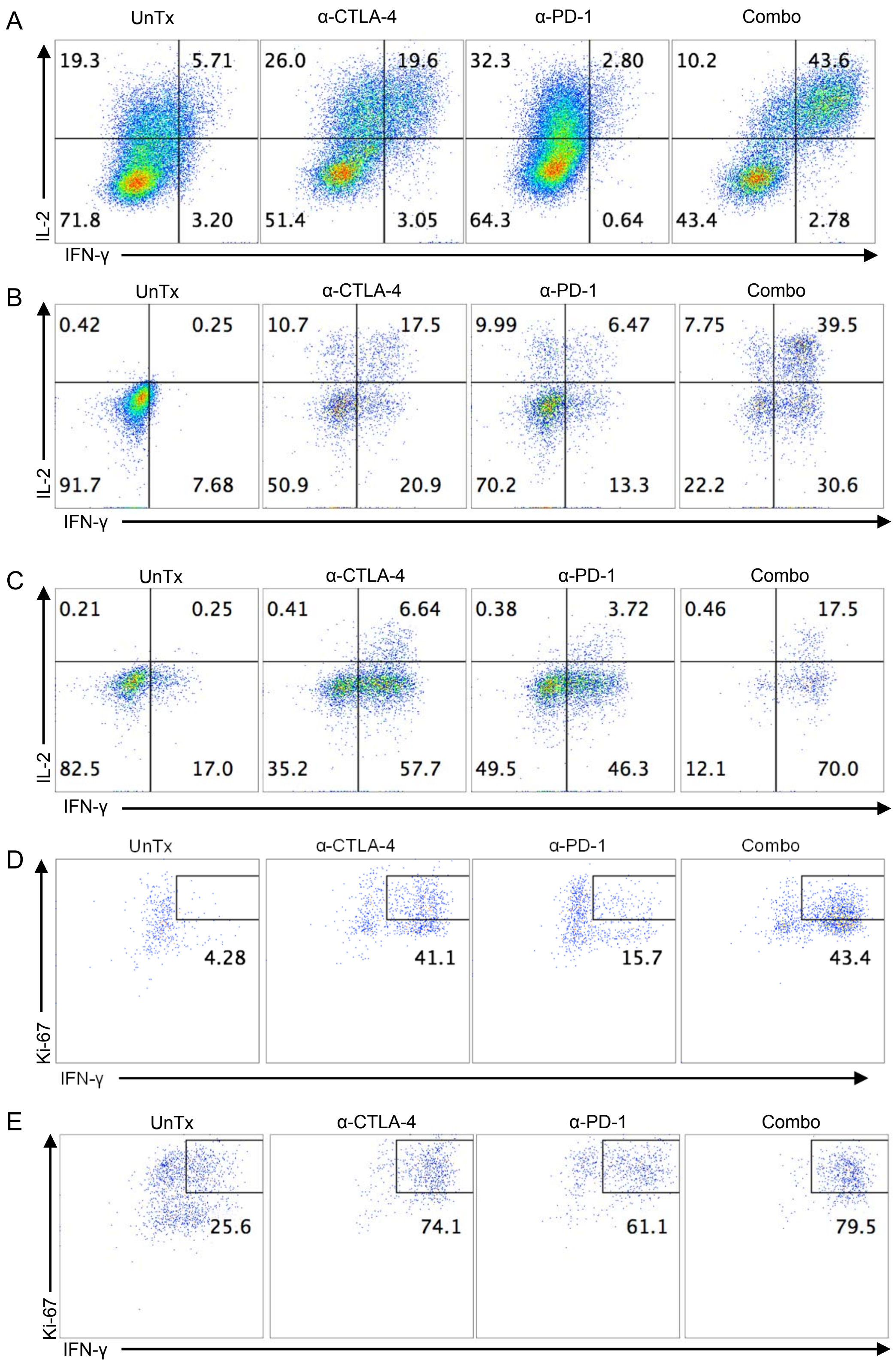

Supplementary Figure 3. Combination therapy boosts polyfunctionality of TILs. (A) Purified CD8$^+$ TILs described as in Fig. 3 co-cultured with irradiated MB49 cells and splenic dendritic cells for 18 h *in vitro* were analyzed for intracellular IL-2 and IFN-γ. (B-E) total TILs from tumor-bearing mice treated with α-CTLA-4-, α-PD-1-, or combo were briefly stimulated with PMA and ionomycin *in vitro*, and production of IFN-γ and IL-2 in CD4$^+$ (B) or in CD8$^+$ (C), as well as expression of IFN-γ and Ki-67 in CD4$^+$ (D) or CD8$^+$ (E) were analyzed by flow cytometry. Data are representative of two independent experiments.

Supplementary Fig. 4

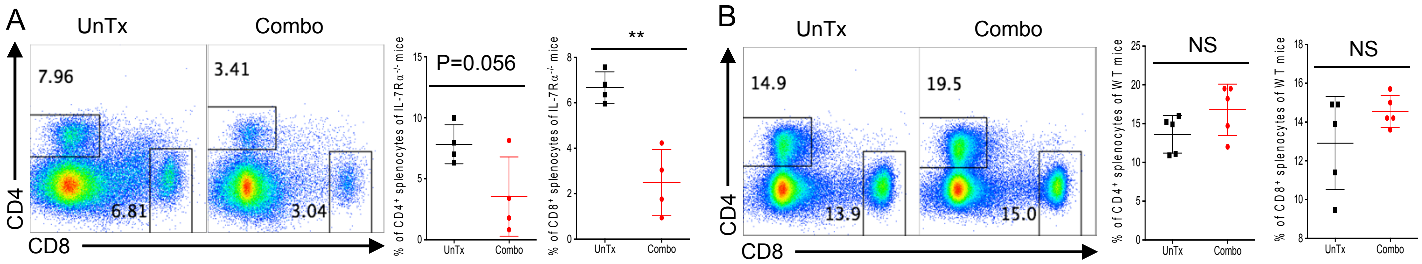

Supplementary Figure 4. Combination therapy reduces frequencies of splenic T cells in *Il7r*$^{-/-}$ tumor-bearing mice. Frequencies of CD4^{+} and CD8^{+} T cells in the spleens of tumor-bearing *Il7r*$^{-/-}$ (A) and WT (B) mice that were untreated (UnTx) or treated with combination therapy (Combo). Pooled results from 4 or 5 mice are shown by the scatter dot plots. Data are means ± SEM. NS, no statistical significance; **, $p<0.01$ by two-tailed unpaired Student's *t*-test. Data are representative of two experiments.

Supplementary Fig. 5

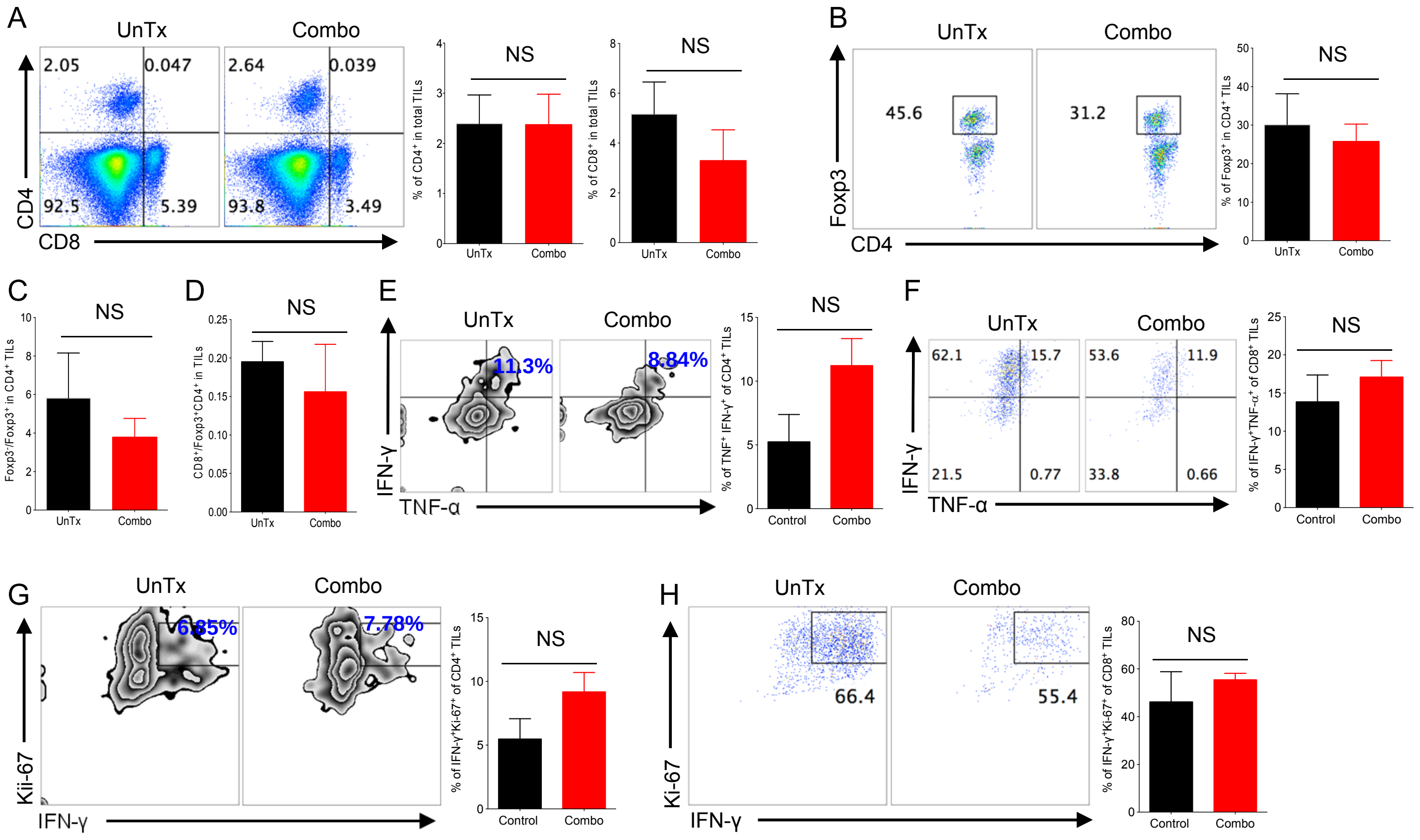

Supplementary Figure 5. IFN-γ signaling is required for combination therapy-induced immune responses. Freshly isolated TILs from *Ifngr1*$^{-/-}$ MB49 tumor-bearing mice untreated (UnTx) or treated with combination therapy (Combo) were directly examined for (A) $CD4^+$ and $CD8^+$ T cell infiltration, (B) Proportions of $Foxp3^+$ T_{reg} among $CD4^+$ TILs, (C) $Foxp3^-/Foxp3^+$ ratios of $CD4^+$ TILs, and (D) $CD8^+/Foxp3^+CD4^+$ ratios. Production of IFN-γ and TNF-α by $CD4^+$ (E) or $CD8^+$ (F) TILs, or expression of Ki-67 and IFN-γ of $CD4^+$ (G) or $CD8^+$ TILs (H) was assessed after a brief stimulation with PMA and ionomycin. Data are means ± SEM of 5 mice in each group. NS, no statistical significance by two-tailed unpaired Student's *t*-test. Data are representative of two independent experiments.

Supplementary Fig. 6

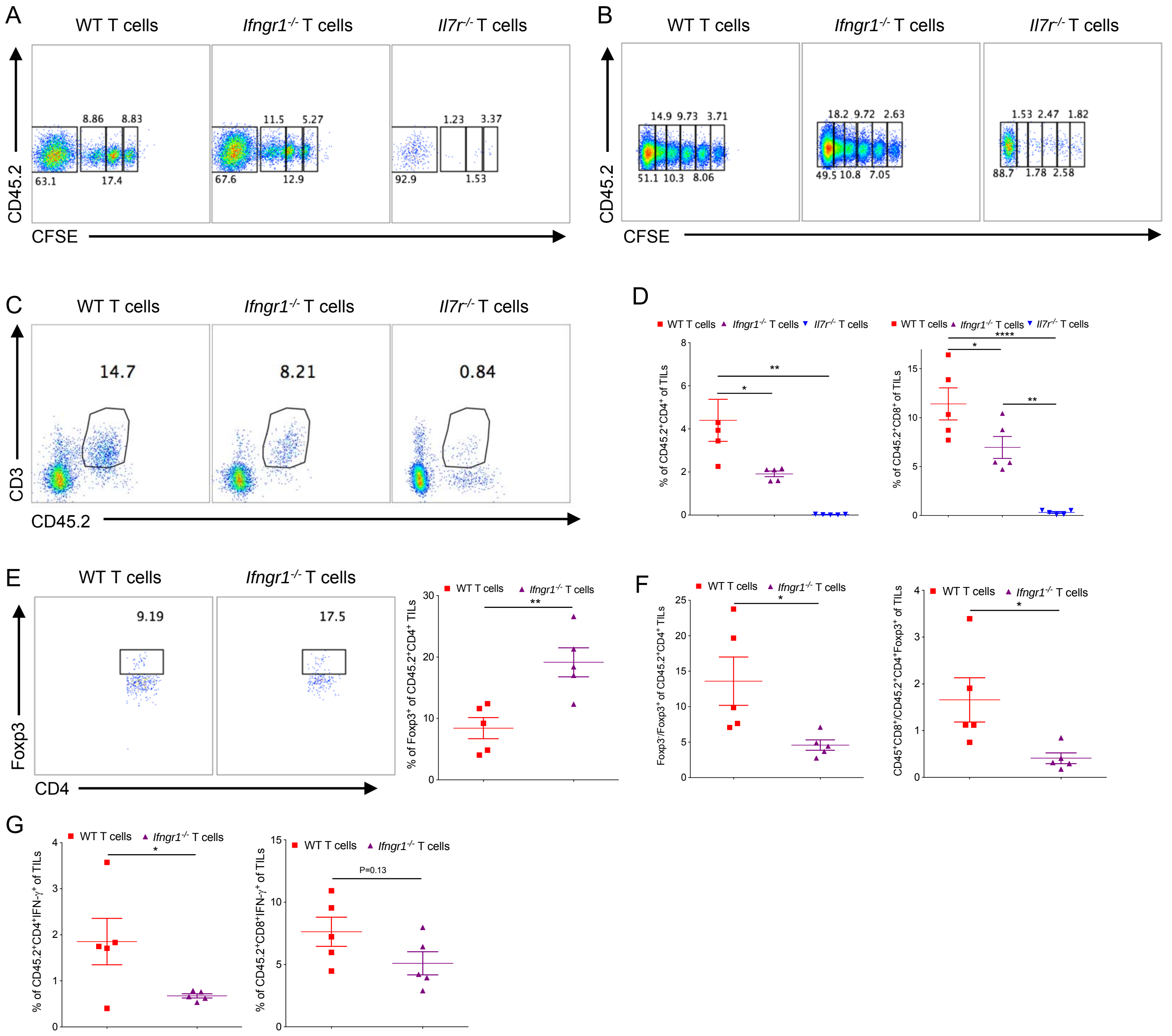

Supplementary Figure 6. IFN-γ and IL-7 signaling in T cells is required for combination therapy-boosted antitumor immunity. CFSE-labeled purified WT, *Ifngr1*$^{-/-}$, or *Il7r*$^{-/-}$ total T cells, congenically marked with CD45.2 were transferred into sublethally irradiated CD45.1 mice, followed by MB49 tumor inoculation and combination therapy. (A-B) CFSE dilution in the CD45.2^{+}CD4^{+} (A) and CD45.2^{+}CD8^{+} (B) splenocytes. Numbers adjacent to gates indicate %. (C) Abundance of transferred T cells (CD45.2^{+}CD3^{+}) in TILs. (D) Frequencies of transferred CD45.2^{+}CD4^{+} and CD8^{+} T cells in TILs. (E) Proportions of T_{reg} among CD45.2^{+}CD4^{+} TILs. Pooled results are presented in the scatter dot plots. (F) Ratios of Foxp3^{-} /Foxp3^{+} in CD45.2^{+}CD4^{+} TILs or CD45.2^{+}CD8^{+}/CD45.2^{+}CD4^{+}Foxp3^{+}CD4^{+} of TILs. (G) Percentages of CD45.2^{+}CD4^{+}IFN-γ^{+} or CD45.2^{+}CD8^{+}IFN- γ^{+} among total TILs. Data are means ± SEM of 5 mice in each group. *, $p<0.05$; **, $p<0.01$; ****, $p<0.0001$ by one-way ANOVA with Bonferroni's *post hoc* test (D) or two-tailed unpaired Student's *t*-test (E-G). Data are representative of two independent experiments.

Supplementary Fig. 7

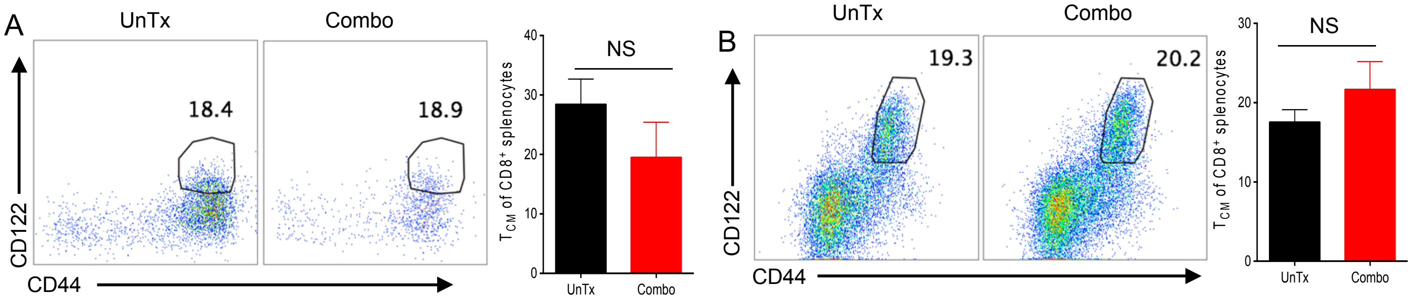

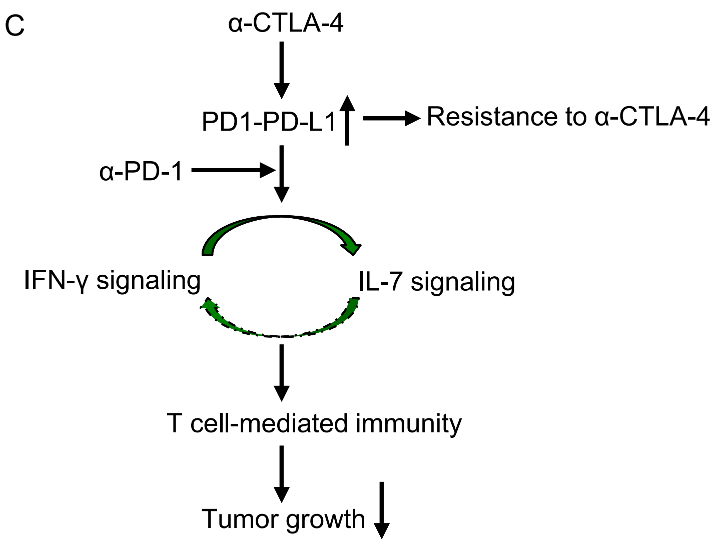

Supplementary Figure 7. Combination therapy does not induce expansion of endogenous CD8$^+$CD44^{hi}CD122$^+$ (T_{CM}) cells in *Il7r*$^{-/-}$ (A) and *Ifngr1*$^{-/-}$ mice (B). Pooled results from 5 mice are depicted as bar graphs. NS, no statistical significance by two-tailed unpaired Student's *t*-test. Data are representative of two independent experiments. (C) A proposed model of how combination therapy exerts therapeutic effects on tumor eradiation by engaging the interdependent loop of IFN-γ and IL-7 signaling in T cells to boost anti-tumor immune responses.
